# An Efficient Distributed Area Division Method for Cooperative Monitoring Applications with Multiple UAVs

**DOI:** 10.3390/s20123448

**Published:** 2020-06-18

**Authors:** José Joaquín Acevedo, Ivan Maza, Anibal Ollero, Begoña C. Arrue

**Affiliations:** GRVC Robotics Lab, University of Seville, Escuela Superior de Ingenieros, Avenida de los Descubrimientos s/n, 41092 Seville, Spain; imaza@us.es (I.M.); aollero@us.es (A.O.); barrue@us.es (B.C.A.)

**Keywords:** unmanned aerial vehicles, multi-UAV, monitoring, area division, distributed system, frequency-based approach, coordination variables

## Abstract

This article addresses the area division problem in a distributed manner providing a solution for cooperative monitoring missions with multiple UAVs. Starting from a sub-optimal area division, a distributed online algorithm is presented to accelerate the convergence of the system to the optimal solution, following a frequency-based approach. Based on the “coordination variables” concept and on a strict neighborhood relation to share information (left, right, above and below neighbors), this technique defines a distributed division protocol to determine coherently the size and shape of the sub-area assigned to each UAV. Theoretically, the convergence time of the proposed solution depends linearly on the number of UAVs. Validation results, comparing the proposed approach with other distributed techniques, are provided to evaluate and analyze its performance following a convergence time criterion.

## 1. Introduction

Gathering and exchanging data among wearable devices, vehicles, sensors, computers and even unmanned aerial vehicles (UAVs) has become crucial to make data accessible instantaneously to make efficient decisions. However, connectivity among interconnected nodes becomes a challenge for long-distance applications because of the communication constraints. In this context, UAVs are playing a relevant role, since they can act as mobile nodes in a wireless sensor network (WSN). They may generate and follow the proper motion plans in order keep communication among all the nodes of the system [1]. In Huang et al. [2], the UAVs move to maximize the quality of coverage considering moving targets, while keeping the connectivity of the network. Other studies have investigated the role of the UAVs as data collectors, which have to plan the proper path in order to visit a set of fixed sensors to gather their data and submit it to the rest of the system [3].

The large area monitoring problem with multiple UAVs is analyzed in this article. Search and rescue [4,5], exploration of disaster areas [6], photogrammetry and mapping [7], precision agriculture [8], intruder detection [9] or inspection of industries [10] are very relevant applications which require the precise monitoring of large areas. Since UAVs can move fast and monitor large areas from the air, this repetitive and heavy task may be performed in a more efficient and safe manner using a coordinated team instead of other type of platforms or manned vehicles.

In particular, this article considers the UAVs as dynamic sensors, able to execute coordinated motion plans, with the objective of continuously collecting and sharing data about the considered area. Assuming restricted communications, the coordinated motion plans must consider at least periodical meetings among the UAVs. This is relevant to keep the connectivity and assure the coordination and data sharing in multi-UAV system. On the other hand, no information is assumed about the location of the events of interest to be detected.

The addressed problem poses several challenging topics that must be taken into account to design the solution. First, it is necessary to define the criteria to optimize during the monitoring mission. This is closely related to the cooperative strategy that the multi-UAV system should implement to monitor the area. Depending on this strategy, a path planning algorithm may be required, such that each UAV can perform its assigned tasks. Moreover, the multi-UAV system must converge to the chosen common strategy in a distributed manner, considering the communication constraints.

This paper addresses explicitly the cooperative patrolling problem for area monitoring missions with multiple UAVs, following a frequency-based approach and under limited communication ranges. In particular, it is focused on the distributed area division problem. The main contribution of this work is the development of an online distributed algorithm to achieve an optimal area partitioning strategy. Starting from an initial sub-optimal area division, the presented solution allows the UAVs to converge to a common and optimal area partitioning strategy, while keeping periodical communications among them. The distributed coordination approach is based on the “coordination variables” concept and a strict neighborhood relation to share information.

## 2. Related Work

Multi-agent area monitoring is a well-studied problem in the literature and it has been addressed following different approaches. The following three key aspects are considered in our paper: the cooperative strategy to cover the area assuming limited communications, the coverage path planning and the distributed coordination method to implement the chosen strategy and follow the planned path.

### 2.1. Cooperative Coverage Strategy

The cooperative strategy chosen to face the area coverage problem will be linked to the available information about the event or phenomena of interest. Assuming a set of specific targets or a known probability distribution about the events of interest, a function to measure the “quality of coverage” may be defined and used as optimization criteria [11,12]. In these cases, the cooperative strategy will consist on the deployment of the UAVs in order to maximize this optimization function but keeping the continuous connectivity of the wireless network, even when some regions of the given area may remain uncovered.

If there is no previous information or models about the phenomena to monitor, it may be assumed a uniform probability distribution of the events of interest through the whole area. In Savkin et al. [13,14], the authors study the minimum number of UAVs, as well as their location, to cover continuously a given area while keeping the connectivity of the system.

However, the number of UAVs and their capabilities may be limited and not enough to cover the whole area. In these cases, a frequency-based approach seems to be the most appropriate way to maximize the probability of detecting the events of interest. The objective is to maximize the frequency of visits to any position in the area [15], which is equivalent to minimize the elapsed time between each pair of consecutive visits to any position in the area [16].

Different deterministic strategies may be adopted to patrol the area maximizing the frequency of visits. Some authors propose team formation techniques to solve the area monitoring problem, where the formation of robots behaves as a single one with larger coverage capabilities [17], following a unique coverage path. A cyclic strategy is adopted in Pasqualetti et al. [18,19], where all the agents track the same closed coverage path, but in the same direction and equally spaced. The authors show that this solution offers theoretically the optimal performance following a frequency-based approach and assuming homogeneous agents (with the same capabilities). However, this solution cannot guarantee the information sharing among the agents under communication constraints and cannot exploit different capabilities with heterogeneous agents.

Partitioning strategies can face these limitations by dividing the whole monitoring mission into sub-area monitoring tasks and forcing periodical meetings between neighbor UAVs [20]). A path partitioning strategy is proposed in Acevedo et al. [21] to monitor an irregular area using heterogeneous UAVs. A unique path is defined to cover the given area and divided into sub-paths to be assigned among the UAVs, being their lengths proportional to the speeds of the UAVs. Each UAV executes “back and forth” motions to patrol its assigned segment. However, this path partitioning strategy cannot get optimal performance following a frequency-based approach, since the end positions of the segments are less frequently visited than the middle positions.

On the other hand, the area partitioning strategies can obtain theoretically the optimal performance, considering UAVs with different capabilities and keeping periodical connectivity [22,23]. This strategy consists on dividing the given area into as many sub-areas as UAVs, being the sub-area size proportional to their covering capabilities. Then, each UAV may generate an only closed path to cover its assigned sub-area.

### 2.2. Coverage Path Planning

With respect to the coverage path planning, the objective is to generate an efficient path to cover a given area, such that any position in the area may be monitored by at least one position in the path [24]. The “Boustrophedon Cellular Decomposition” [25] is the best-known coverage path planning algorithm. It proposes to divide the whole area into smaller sub-areas, which can be covered with a simple “back and forth” path. Other authors propose to generate a spanning tree based on the “Approximated Cellular Decomposition” and to build the coverage path as its boundary [26]. A different approach based on gradient fields and neural networks is presented in Yang et al. [27]. These algorithms are focused on the coverage path planning considering the presence of obstacles. However, our paper addresses specifically the multi-path planning for area covering path, addressing the synchronization among them. In Guruprasad et al. [28], an in-line algorithm based on “Voronoi spatial partitioning” is proposed to generate coverage paths for multiple robots in a distributed manner. On the other hand, in Acevedo et al. [23], it is proposed a planning algorithm to generate closed coverage paths for irregular areas which assures equal path length between each pair of consecutive given positions, ensuring synchronization between multiple aerial robots.

### 2.3. Distributed Coordination

Regarding the distributed coordination of multiple agents to converge to a common strategy, the “coordination variables and coordination functions theory” claims that a common cooperative behavior may be achieved in a distributed manner using a limited set of variables [29]. If all the UAVs share these coordination variables and apply their own coordination function, the whole system will converge to a common solution.

A coordination variables approach was applied in Kingston et al. [30] to coordinate a set of UAVs to adopt a path partitioning strategy for perimeter monitoring missions, assuming communication constraints. However, our paper addresses a much more complex and coupled problem, the distributed coordination of multiple UAVs to implement an area partitioning strategy. In particular, our paper considers how each UAV may decide its assigned sub-area in a distributed manner, taking into account that its shape must be consistent with the assigned to the rest of UAVs. Moreover, the synchronization to share information between neighbor UAVs should be addressed in the solution. Previous works have proposed simplified versions which basically try to divide the whole problem into sub-problems which may be more easily solved based on a coordination variables approach.

The “one-to-one coordination” technique considers a simple and independent coordination problem for each pair of agents. This technique is proposed to solve the distributed area allocation problem among a set of UAVs in Acevedo et al. [31] converging to the optimal area partitioning strategy. To accelerate the convergence, the “block-sharing technique” was introduced in Caraballo et al. [32] which extends the coordination problem from pair of agents to larger groups. Each UAV waits to receive individual information from the rest of UAVS which belong to the same block, and then solves the area division. Finally, the “column-row decoupled algorithm”, introduced in Acevedo et al. [33], proposes to solve the two-dimensions area division problem as two decoupled one-dimension division problems following similar techniques to these proposed in Kingston et al. [30] for each one. These methods have shown to converge successfully to the area partitioning strategy in a distributed manner.

The coordination of multiple UAVs to adopt a common area patrolling strategy in a distributed manner, assuming not continuous but intermittent communications, is the main challenge addressed in this paper. This paper proposes a “coordination variables” approach to reduce the convergence time even for large-scale problems and to reduce the required information storage capability.

## 3. Area Monitoring with Multiple UAVs Following a Frequency-Based Approach

**Figure 1:**
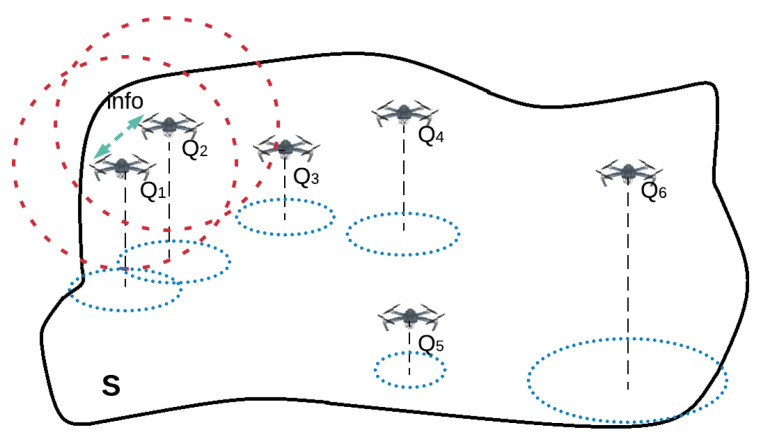
A team of 6 UAVs in charge of monitoring a given area in a cooperative manner.

Given a large area defined by a polygon **S** of size *A*, a team of *n* UAVs $Q:=\{Q_{1},Q_{2},\ldots ,Q_{n}\}$ has to monitor it following a frequency-based approach, see Figure 1. Onwards, the following assumptions are considered about the UAVs:

- The UAVs are equipped with the appropriate sensors to detect the edges of the area **S**.
- Communication constraints are considered. A pair of UAVs may communicate if and only if they are within their communication range *R*, which is small compared to the size of the area **S**, i.e., R<<A.
- For each UAV $Q_{i}$, the sensing coverage $a_{i}$ is defined as the size of area that it can monitor each unit of time, being the more relevant parameter to address the problem. It integrates other parameters such as flying speed, coverage range, etc.
- The sub-area assigned to each UAV $Q_{i}$ at any time *t* is defined as $S_{i}\left(t\right)$. The initial area division, defined by $S_{i}\left(0\right)$, ∀i=1…n, includes the whole area **S**.

**Figure 2:**
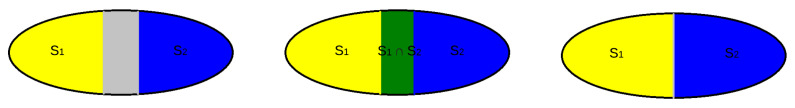
Area division between two identical agents. Yellow, blue and gray indicate the area assigned to the first UAV, the second UAV and unassigned, respectively. Image on the left shows a sub-optimal division where $S_{1}\cup S_{2}\neq S$. Image in the middle shows a sub-optimal division because $S_{1}\cap S_{2}\neq \emptyset$. Image on the right shows an optimal area division matching the conditions shown in Equation (1).

To maximize the frequency of visits (or minimize the elapsed time between consecutive visits) to any position in **S**, an area partitioning strategy is posed to organize the *n* UAVs. An optimal area partitioning strategy implies that each UAV has assigned a non-overlapping sub-area $S_{i}^{\prime }$ with a size according to its sensing coverage, such that the union of them is **S** (see Equation (1) and Figure 2).

(1) $$\begin{array}{c} \begin{array}{c} A_{i}^{\prime }:=a_{i}\frac{A(S)}{\sum_{j=1}^{n}a_{j}}\\ \overset{n}{\underset{i=1}{⋂}}S_{i}^{\prime }=\emptyset \\ \overset{n}{\underset{i=1}{⋃}}S_{i}^{\prime }=S \end{array} \end{array}$$

where A(X) is a function which returns the size of an area defined by a polygon **X** given as input. Then, according to the problem description, A(S)=A.

**Figure 3:**
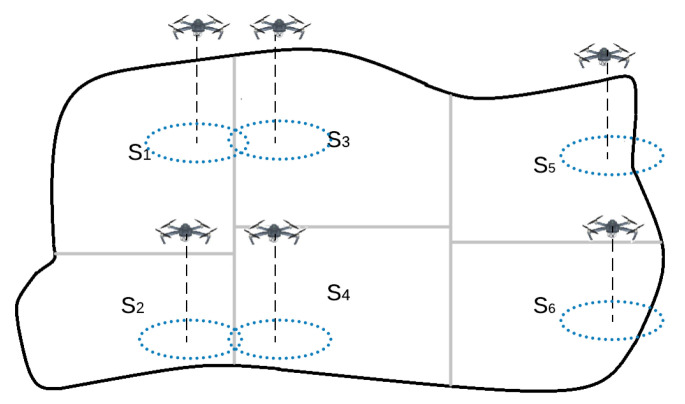
The area is divided into 6 non-overlapping sub-areas which are assigned to the different UAVs.

Therefore, the objective is to find a distributed algorithm which accelerates the convergence from an initial sub-optimal area partitioning strategy, defined by the initial assigned sub-areas ($S_{i}\left(0\right)$, ∀i=1…n), to a final distribution (see Figure 3) ($S_{i}\left(t\right)$, ∀i=1…n) which fulfils the conditions defined by

(2) $$\begin{array}{c} \begin{array}{c} \max_{i}\left(\right|A_{i}^{\prime }-A\left(S_{i}\left(t\right)\right)\left|\right)<\epsilon \\ \overset{n}{\underset{i=1}{⋂}}S_{i}\left(t\right)=\emptyset \\ \overset{n}{\underset{i=1}{⋃}}S_{i}\left(t\right)=S \end{array} \end{array}$$

being ϵ>0 defined as the maximum tolerance.

## 4. Distributed Area Allocation Based on Coordination Variable

Communications restriction is a key component to consider in the design of an efficient method for area partitioning with a set of multiple UAVs in a distributed manner. Moreover, UAVs will require the sharing of information about the monitoring scenario: capabilities of the UAVs, size and shape of the assigned sub-areas, detected events, etc. Therefore, meetings among the UAVs have to be forced structuring the multi-UAV system as a communication grid where each UAV has between one and four neighbors and a predefined list of positions $M_{i}$ to meet them. This list includes the following meeting positions: $m_{i}^{above}$, $m_{i}^{below}$, $m_{i}^{left}$ and $m_{i}^{right}$.

Coordination variables theory claims that for any multi-agent coordination problem, there is a limited set of variables which should be shared by all the agents to solve the problem in a distributed manner. If all the UAVs have the same values for these variables, all the UAVs will converge to the same solution in an independent manner. Then, it is relevant to choose properly the coordination variables. In this case, the chosen ones have been the area to monitor $A_{t}=A\left(S\right)$ and the sum of all the covering speeds of the UAVs $a_{t}=\sum_{i=1}^{n}a_{i}$. Equation (1) shows that using those values, each UAV could calculate its optimal assigned area to accomplish an area partitioning strategy.

**Figure 4:**
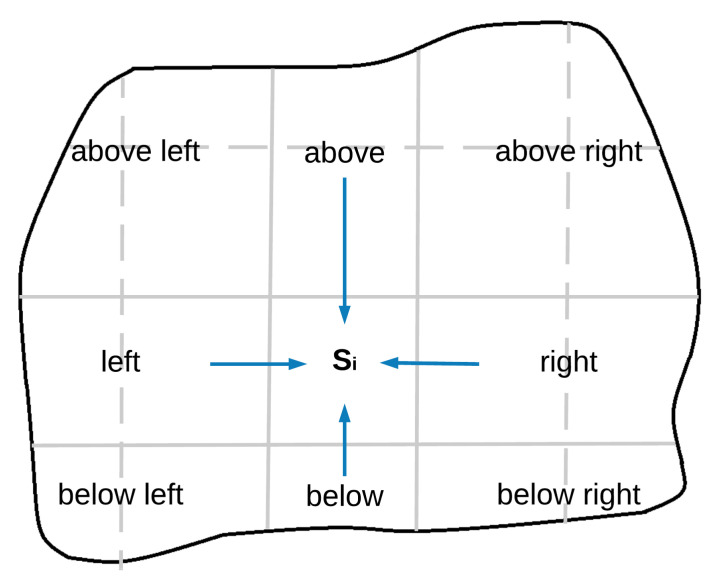
Area division and grouping depending on the side with respect to the sub-area assigned to the UAV $Q_{i}$, considering a 4×5 grid configuration.

However, the problem is not only to decide the size of area to be assigned to each UAV, but also the shape and position of that sub-area, so that they match the conditions specified in Equation (1). The proposed solution organizes the division of the area as a 2D grid, where each sub-area is related to the rest according to its column and row. Each UAV $Q_{i}$ groups the sub-areas and UAVs into nine sides (see Figure 4). Then, it defines a set of intermediate variables to model these sides: the polygons which define the areas ($S_{i}$, $S_{i}^{l}$, $S_{i}^{al}$, $S_{i}^{a}$, $S_{i}^{ar}$, $S_{i}^{r}$, $S_{i}^{br}$, $S_{i}^{b}$, $S_{i}^{bl}$) and the sum of covering speeds ($a_{i}$, $a_{i}^{l}$, $a_{i}^{al}$, $a_{i}^{a}$, $a_{i}^{ar}$, $a_{i}^{r}$, $a_{i}^{br}$, $a_{i}^{b}$, $a_{i}^{bl}$) on each side. Superscripts al, a, ar, l, r, bl, b and br indicate that the considered variable refers to the side above left, above, above right, left, right, below left, below and below right, respectively.

These intermediate variables may be updated using the information received from neighboring UAVs and depending on their relative position (above, below, right and left). Based on these variables, each UAV can compute the coordination variables to define the whole scenario. Moreover, the intermediate variable will be necessary to implement a coherent area division which matches the conditions defined in Equation (1) in a distributed manner, according to the protocol explained in Section 4.1.

**Figure 5:**
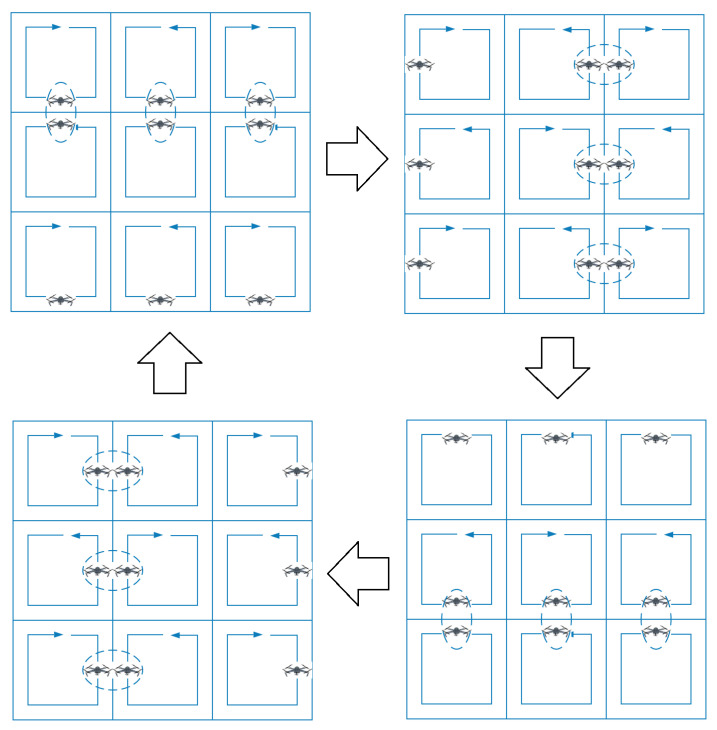
A synchronized multi-UAV system performing an area partitioning strategy, where neighbor UAVs patrol their closed paths in opposite directions. Each UAV meets periodically with each of its neighbors.

Each UAV $Q_{i}$ knows its covering speed $a_{i}$, its initially assigned sub-area $S_{i}$ and initializes the rest of its intermediate variables to 0 (covering speeds) or ∅ (areas). Then each UAV generates a closed path to patrol its assigned area $S_{i}$ based on the path planning method proposed in Acevedo et al. [23]. This path visits the list of positions $M_{i}$, such that the length between each pair of consecutive positions is always the same. The generated path may achieve periodic communications among neighbor UAVs, since all the UAVs will take the same time to travel between two consecutive meeting positions. The objective is to synchronize the whole multi-UAV system, while maximizing the frequency criterion. Please note that neighbor UAVs must follow their paths in opposite directions to achieve synchronization among them, see Figure 5.

Then, the UAVs take off and start following their own paths. When a UAV $Q_{i}$ reaches a position $m_{i}^{side}$ included in $M_{i}$, it waits to meet its neighbor $Q_{j}$ to share and update the appropriate intermediate variables. These intermediate variables must be updated by $Q_{i}$ and $Q_{j}$, depending on their relative positions. If $Q_{i}$ is the left neighbor of $Q_{j}$, $Q_{i}$ will update its intermediate variables using information provided by $Q_{j}$, according to Equation (3). Similarly, $Q_{j}$ will update its intermediate variables using information received from $Q_{i}$, according to Equation (4).

(3) $$\begin{array}{c} \begin{array}{c} S_{i}^{r}\leftarrow S_{j}^{r}\cup S_{j}\\ S_{i}^{ar}\leftarrow S_{j}^{ar}\cup S_{j}^{a}\\ S_{i}^{br}\leftarrow S_{j}^{br}\cup S_{j}^{b}\\ a_{i}^{r}\leftarrow a_{j}^{r}+a_{j}\\ a_{i}^{ar}\leftarrow a_{j}^{ar}+a_{j}^{a}\\ a_{i}^{br}\leftarrow a_{j}^{br}+a_{j}^{b} \end{array} \end{array}$$

(4) $$\begin{array}{c} \begin{array}{c} S_{j}^{l}\leftarrow S_{i}^{l}\cup S_{i}\\ S_{j}^{al}\leftarrow S_{i}^{al}\cup S_{i}^{a}\\ S_{j}^{bl}\leftarrow S_{i}^{bl}\cup S_{i}^{b}\\ a_{j}^{l}\leftarrow a_{i}+a_{i}^{l}\\ a_{j}^{al}\leftarrow a_{i}^{al}+a_{i}^{a}\\ a_{j}^{bl}\leftarrow a_{i}^{bl}+a_{i}^{b} \end{array} \end{array}$$

However, if $Q_{i}$ is the neighbor below $Q_{i}$, they will update their intermediate variables sharing information according to the Equations (5) and (6), respectively.

(5) $$\begin{array}{c} \begin{array}{c} S_{i}^{a}\leftarrow S_{j}^{a}\cup S_{j}\\ S_{i}^{ar}\leftarrow S_{j}^{ar}\cup S_{j}^{r}\\ S_{i}^{al}\leftarrow S_{j}^{al}\cup S_{j}^{l}\\ a_{i}^{a}\leftarrow a_{j}^{a}+a_{j}\\ a_{i}^{ar}\leftarrow a_{j}^{ar}+a_{j}^{r}\\ a_{i}^{al}\leftarrow a_{j}^{al}+a_{j}^{l} \end{array} \end{array}$$

(6) $$\begin{array}{c} \begin{array}{c} S_{j}^{b}\leftarrow S_{i}^{b}\cup S_{i}\\ S_{j}^{br}\leftarrow S_{i}^{br}\cup S_{i}^{r}\\ S_{j}^{bl}\leftarrow S_{i}^{bl}\cup S_{i}^{l}\\ a_{j}^{b}\leftarrow a_{i}+a_{i}^{b}\\ a_{j}^{br}\leftarrow a_{i}^{br}+a_{i}^{r}\\ a_{j}^{bl}\leftarrow a_{i}^{bl}+a_{i}^{l} \end{array} \end{array}$$

Once each UAV $Q_{i}$ has updated its intermediate variables, it can recompute its coordination variables according to Equation (7). The polygon which defines the whole area to monitor by the multi-UAV system is built as the union of the polygons which define each side sub-area. The covering capabilities of the whole multi-UAV system is featured by the sum of the covering capabilities of the UAVs located at each side. As each UAV receives more information from its neighbors, these two values tend to the actual values which define the problem (**S** and $\sum_{i=1}^{n}a_{i}$, respectively). Using these coordination variables, each UAV may decide its own assigned sub-area $S_{i}$ following the protocol described in Section 4.1.

(7) $$\begin{array}{c} \begin{array}{c} A_{t}\leftarrow A\left(S_{i}\cup S_{i}^{l}\cup S_{i}^{al}\cup S_{i}^{a}\cup S_{i}^{ar}\cup S_{i}^{r}\cup S_{i}^{br}\cup S_{i}^{b}\cup S_{i}^{bl}\right)\\ a_{t}\leftarrow a_{i}+a_{i}^{l}+a_{i}^{al}+a_{i}^{a}+a_{i}^{ar}+a_{i}^{r}+a_{i}^{br}+a_{i}^{b}+a_{i}^{bl} \end{array} \end{array}$$

Then, each UAV updates the meeting position $m_{i}^{side}$ to the nearest point in the edge of its new assigned sub-area and recomputes a closed coverage path using the method proposed in Acevedo et al. [23]. This process is repeated until the mission is aborted.

### 4.1. Area Division Protocol

The proposed area division protocol is based on the communication grid structure of the multi-UAV system. Let us assume that the grid has *r* rows and *c* columns, such that n=r·c. The proposed protocol implies first to divide the whole area in *c* sub-areas using vertical lines, according to the sum of covering speeds of the UAVs of each column. In addition, then, each column sub-area is divided in *r* sub-areas using horizontal lines and based on the individual covering speeds of the UAVs. To apply this protocol, in a distributed manner, the intermediate and coordination variables are used.

**Figure 6:**
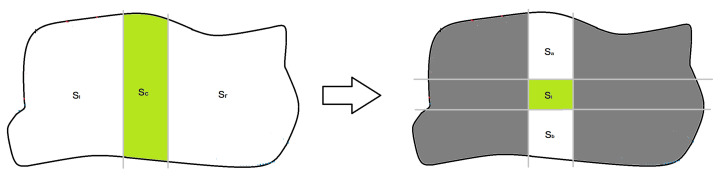
Area division protocol used by each UAV $Q_{i}$ to self-assign its own sub-area $S_{i}$. For each step, gray lines are used to make divisions and the green polygon is the chosen one.

The process is illustrated in Figure 6. Each UAV $Q_{i}$ computes the sizes of area which should be assigned to all the UAVs in its own column $A_{c}$, on right columns $A_{r}$ and on its left columns $A_{l}$ as

(8) $$\begin{array}{c} \begin{array}{c} A_{c}\leftarrow \left(a_{i}+a_{i}^{a}+a_{i}^{b}\right)\frac{A_{t}}{a_{t}}\\ A_{r}\leftarrow \left(a_{i}^{r}+a_{i}^{ar}+a_{i}^{br}\right)\frac{A_{t}}{a_{t}}\\ A_{l}\leftarrow \left(a_{i}^{l}+a_{i}^{al}+a_{i}^{bl}\right)\frac{A_{t}}{a_{t}} \end{array}. \end{array}$$

Using vertical lines, and according to these values, the area **S** is divided into three sub-areas: $S_{c}$, $S_{l}$ and $S_{r}$.

Now, the size of the areas that should be assigned to the UAVs above ($A_{a}$) and below ($A_{b}$) $Q_{i}$ but in the same column is calculated as follows

(9) $$\begin{array}{c} \begin{array}{c} A_{i}\leftarrow a_{i}\frac{A_{c}}{a_{i}+a_{i}^{a}+a_{i}^{b}}\\ A_{a}\leftarrow a_{i}^{a}\frac{A_{c}}{a_{i}+a_{i}^{a}+a_{i}^{b}}\\ A_{b}\leftarrow a_{i}^{b}\frac{A_{c}}{a_{i}+a_{i}^{a}+a_{i}^{b}} \end{array}. \end{array}$$

Finally, the column sub-area is divided using horizontal lines according to these values and obtaining the sub-area $S_{i}$ to be assigned to $Q_{i}$.

### 4.2. Analysis of Convergence

**Figure 7:**
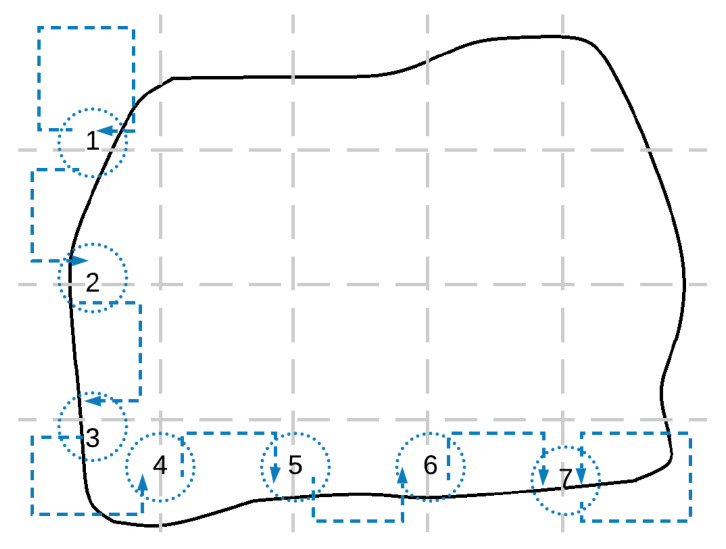
Required peer-to-peer communications to share an information between the two farthest UAVs, considering a 4×5 grid configuration.

The convergence time is related to the time in which each UAV receives the information from the rest of the UAVs, even in an indirect way. Let us assume that *n* UAVs have divided the considered area into *n* sub-areas, according to a r×c grid, to allow periodic connectivity. Then, n=r·c. The maximum number of sub-areas (cells in the grid) between the two farthest UAVs is r+c−1, not *n*. Therefore, the convergence time will depend linearly on r+c−2, as illustrated in Figure 7.

## 5. Validation Results

More than 100 MATLAB simulations have been performed to validate the proposed solution and analyze the convergence time depending on the number of UAVs and the grid configuration.

Each simulation defines a different scenario considering irregular areas, a r×c grid configuration, non-efficient initial area division and a set of random parameters for the UAVs. Then, this scenario is simulated using the proposed algorithm based on coordination variables. To compare the results, the same scenarios are also simulated implementing algorithms based on other approaches: the one-to-one coordination method [31], the block-sharing strategy [32] and the column-row decoupled algorithm [33]. For the block-sharing strategy, a 4×4 block is considered. Each simulation runs until conditions specified in Equation (2) are met assuming an ϵ=A/100, being *A* the size of the considered area.

### 5.1. Detailed Results from a Particular Scenario

**Figure 8:**
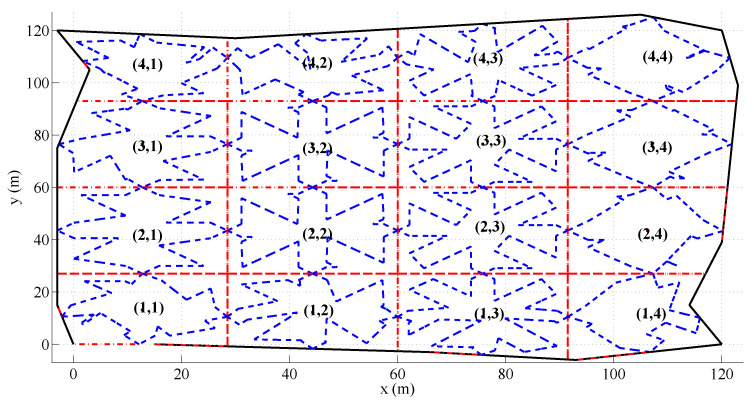
Initial area division considered during the simulations. The solid black lines define the irregular area to monitor. The dashed black lines determine the initial sub-areas assigned to the UAVs and the dashed blue lines their initial coverage paths generated using the path planning method proposed in Acevedo et al. [23].

To better illustrate the simulations performed, detailed results from a particular scenario are described in the following. The scenario considers an irregular area with a size of 15,000 m${}^{2}$, which has to be monitored by 16 UAVs with different sensing capabilities (see Table 1), following an area partitioning strategy. They are organized according to a 4×4 grid configuration. The different algorithms have been tested starting from an initial non-efficient area division shown in Figure 8.

**Figure 9:**
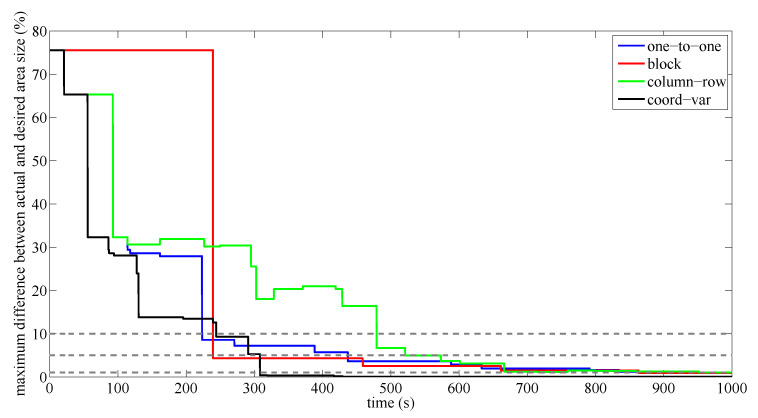
Evolution along the time of the maximum difference between the assigned area and the optimal one, using different coordination methods. The gray dashed lines determine a maximum relative difference of 10%, 5% and 1%

To analyze how the actual area division tends to the optimal one following a frequency-based approach, the maximum difference between the size of the sub-areas currently assigned to the UAVs and the optimal ones (as they are defined in Equation (1)) is computed along time. Results are shown in Figure 9.

These results show that the proposed solution based on coordination variables converges faster than other approaches to the optimal solution, assuming ϵ=A/100. The trend of the solution obtained using the proposed method keeps almost constant to the optimal division. Therefore, it is an any-time algorithm since it always provides a better solution while it is running. Meanwhile, the improvement of the solution based on the one-to-one coordination is fast at the beginning (until 10%), but slows down after. Therefore, obtaining a very accurate area division, according to the optimal one defined by Equation (2), may be very slow. Regarding the block-sharing strategy, the results show that this method requires larger slots of time to update the area division. Then, the initial sub-optimal division remains more time, but the improvement of the solution is higher. Anyway, the time required to get accurately a solution close to the optimal is significantly worse than using the proposed method. Otherwise, the solution provided by the column-row decoupled approach is irregular and much worse than the others, showing it as a no any-time algorithm.

**Figure 10:**
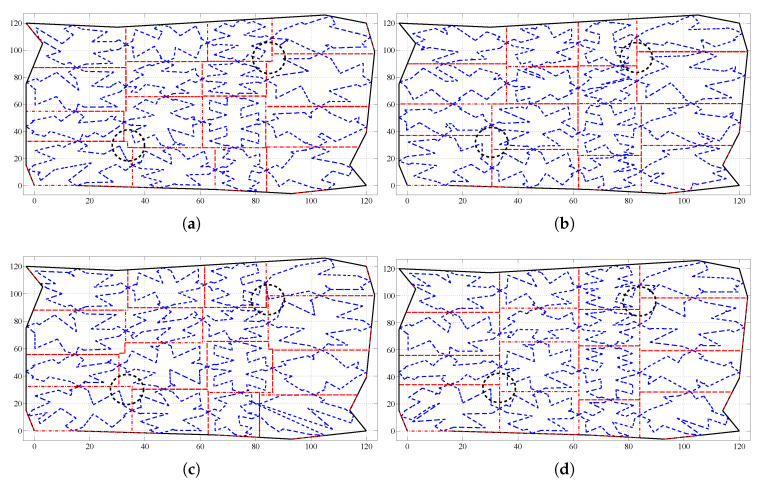
Final area division obtained during the simulation using the coordination algorithms: (**a**) based on the one-to-one coordination, and (**b**) based on the (2,2)-block-sharing strategy. (**c**) based on the column-row decoupling, and (**d**) based on the coordination variables. The dashed red lines determine the final sub-area shapes assigned to the UAVs, the dashed blue lines indicate their coverage paths generated using the path planning method proposed in Acevedo et al. [23] and the thick dashed black circles highlight two examples of the edges of shapes of the computed sub-areas.

Figure 10 shows the final area divisions achieved using the different methods, assuming an epsilon of 1% of the size the whole area.

The proposed algorithm based on the coordination variables gets rectangular shapes for the sub-areas, whereas other algorithms compute sub-areas with more irregular edges. Then path planning problem is simplified and shorter closed paths to cover the whole sub-area are computed.

### 5.2. Convergence Time Analysis

**Figure 11:**
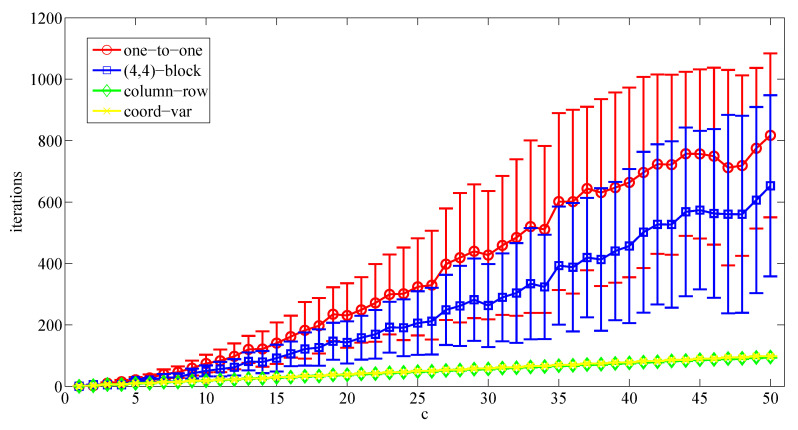
Average number of meetings (±its standard deviation) required to converge to the specified area partition strategy depending on the number of UAVs and considering a 1×c configuration and different coordination algorithms.

A first set of simulations considers that the multi-UAV system is organized according to a 1×c grid configuration (as a vector). It means that all UAVs are connected and each one has at most two neighbors. There is a unique line of communication between each pair of UAVs in the system. Figure 11 shows the number of meetings required by each UAV to converge to the optimal area partitioning strategy, depending on *c*.

According to the obtained results, it seems that the one-to-one and the block-sharing strategies get a similar performance from a convergence time point of view while increasing the number of UAVs. The convergence time achieved using the one-to-one coordination method keeps positive with respect to the block-sharing strategy, which will probably depend on the size of the block. In both cases, the convergence time seems to depend quadratically on the number of UAVs. On the other hand, the proposed solution based on the coordination variables has much better performance than both methods, being its convergence time linearly dependent on the number of UAVs. In these cases, the column-row decoupled solution gets the same performance than the proposed solution. It makes sense since both approaches are similar when the UAVs are connected following a vector configuration.

**Figure 12:**
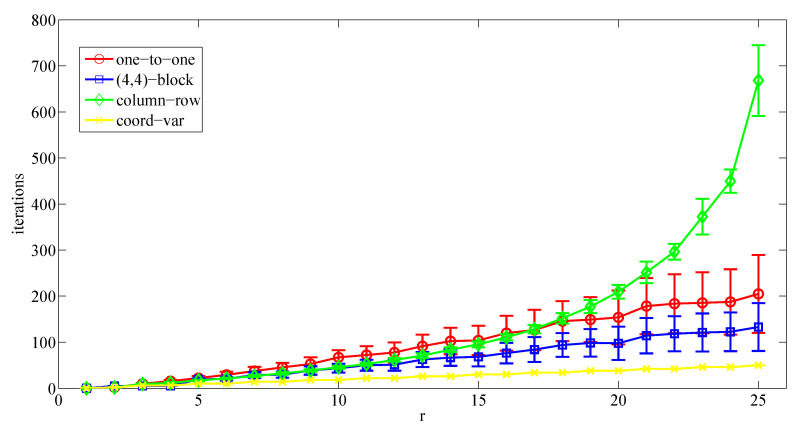
Average number of meetings (±its standard deviation) required to converge to the specified area partition strategy considering a r×r configuration and depending on the number of UAVs and different coordination algorithms.

Then, a second set of simulations considers a r×r grid configuration. This means that the multi-UAV system implements a connectivity graph defined as a square matrix (same number of columns and rows). Therefore, each UAV has up to four neighbors. Figure 12 shows the number of meetings required by each UAV to converge to the optimal area partitioning strategy depending on $r=\sqrt{n}$.

These results confirm the analysis related to the performance of the proposed approach based on the coordination variables and the ones based on the one-to-one and block-sharing strategies. The proposed solution achieves much better performance from a convergence time point of view while increasing the number of UAVs. Finally, the performance of the column-row algorithm gets worse dramatically for these scenarios when the number of UAVs increases.

## 6. Discussion

The cooperative area coverage problem with a team of UAVs can be addressed from different points of view. An interesting approach considers the deployment of the UAVs through the considered area trying to maximize the covered area or to optimize some kind of criteria based on the probability distribution of the events of interest [11,12]. Our method follows a different approach: covering the whole area by implementing a cooperative patrolling strategy. The objective is to minimize the time to cover the whole area. The other approaches cited above cannot be directly compared since they have different objectives. However, the approaches can be complementary. The proposed method requires starting from an initial area division, which may be obtained using a method which maximizes the covered area while keeping the multi-UAV system connectivity.

The area partitioning strategy proposed to patrol cooperatively the area requires an efficient area division. Previous works demonstrate that the time required to cover the whole area may be minimized if the size of the sub-area assigned to each UAV is proportional to its own covering capability.

In large-scale scenarios, communication constraints must be considered, and a distributed solution becomes the most appropriate way to address the problem. Previous works have proposed distributed methods to divide the area, but the solutions converge too slow to the most accurate area division.

Our paper proposes a more efficient solution from a convergence time point of view, since it requires fewer iterations to achieve the optimal area division independently of the size of the area and the grid considered for the division. Results also show that unlike other methods, the proposed algorithm is any-time because it can always provide a valid solution, being the difference between the current and the optimal division decreasing along time. In addition, it converges to the most accurate area division faster than the rest of algorithms and the sub-area assigned to each UAV is more regular compared to other methods, allowing a more efficient covering path to monitor it. Finally, the memory storage requirements for the implementation of the proposed algorithm is similar or lower with respect to the other considered methods.

The addressed problem and the proposed method are explained in this paper based on an irregular area but convex and almost rectangular. Therefore, it is easy to divide the area into several sub-areas following a matrix configuration (see Figure 3). The proposed solution exploits this type of configuration to obtain the minimum convergence time. Anyway, this approach could be applied to other more complex shapes (U-shape, S-shape, Star-shape, polygon with holes, etc.), just defining the most suitable configuration to divide each area shape. This configuration will determine the neighbors of each UAV. However, the performance will depend on the distance between the two farthest UAVs through the connectivity graph.

## 7. Conclusions and Future Work

This paper presents a distributed area division algorithm to coordinate a team of UAVs to monitor cooperatively an area under communication constraints. The proposed system is based on coordination variables and a predefined division protocol to ensure a coherent area allocation. This tends to an optimal solution following a frequency-based approach. The studied problem has been previously addressed applying different algorithms, which offer valid, dynamic and scalable solutions, but have different drawbacks related to their convergence times and memory storage requirements. For instance, block-sharing strategies can get low convergence times but assuming large block sizes, which requires storage of the individual data from each UAV included in the block.

The proposed algorithm extends previous solution for the path partitioning division to the area division problem. It has the advantages of other distributed area division algorithms, and offers a faster convergence rate, low memory storage requirements and an area division which facilitates the generation of efficient coverage paths. The convergence time depends linearly on the number UAVs, but taking into account how they are organized, i.e., if they are organized as a 2D-grid to share information, the convergence time will depend on the sum of columns and rows of that grid. Validation results confirm this analysis and compare the presented approach to others.

Future developments will include non-deterministic cooperative strategies to survey the area for security applications because the posed deterministic strategy could turn to inefficient if it is learned by smart intruders. On the other hand, the proposed system assumes that the coverage path length depends linearly on the area shapes, but this condition should be deeply analyzed. Therefore, online coverage path planning algorithms which maximize the monitored area will be studied. The periodic connectivity of the system should be considered in this study. Finally, the proposed solution should be extended considering a dynamic grid, where their dimensions and even the relative position of the UAVs could change during the mission, in order to get a more robust and fault-tolerant system.

## Figures and Tables

**Table 1:** This table includes the capabilities of the UAVs involved in the studied scenario, according to their location in the grid: flying velocity ($v_{i}$), coverage range ($c_{i}$) and sensing coverage ($a_{i}=2c_{i}v_{i}$).

Grid Index	$v_{i}$ (m/s)	$c_{i}$ (m)	$a_{i}$ (m${}^{2}$/s)
(1,1)	0.95	4.28	8.13
(1,2)	0.83	3.58	4.28
(1,3)	0.66	3	3.96
(1,4)	0.68	5.20	7.07
(2,1)	0.61	4.28	5.22
(2,2)	0.98	3.58	7.02
(2,3)	0.97	3	5.82
(2,4)	0.70	5.20	7.28
(3,1)	0.88	4.28	7.53
(3,2)	0.66	3.58	4.30
(3,3)	0.65	3	3.90
(3,4)	0.95	5.20	9.88
(4,1)	0.77	4.28	6.59
(4,2)	0.75	3.58	5.37
(4,3)	0.78	3	4.68
(4,4)	0.61	5.20	6.34
